# Antibody-recognizing residues 188-211 of TMEM106B exhibit immunohistochemical reactivity with the TMEM106B C-terminal fragment

**DOI:** 10.3389/fnins.2023.1250547

**Published:** 2023-10-23

**Authors:** Ruoyi Ishikawa, Yu Yamazaki, Masahiro Nakamori, Tetsuya Takahashi, Hirofumi Maruyama

**Affiliations:** ^1^Department of Clinical Neuroscience and Therapeutics, Hiroshima University Graduate School of Biomedical and Health Sciences, Hiroshima, Japan; ^2^Department of Rehabilitation, Faculty of Rehabilitation, Hiroshima International University, Hiroshima, Japan

**Keywords:** TMEM106B, C-terminal fragment, fibrils, new antibody, neurodegenerative disease, FTLD-MND

## Abstract

Accumulation of TMEM106B fibrils composed of cleaved C-terminal fragments (CTF) of transmembrane protein 106B (TMEM106B) has recently been observed in the brains of elderly subjects and individuals with neurodegenerative diseases. To date, one antibody recognizing the residues 239-250 has been found to display immunoreactivity to the TMEM106B CTF, thereby defining TMEM106B C-terminal immunoreactive (TMEM-ir) material. Immunohistochemical characterization of the CTF using antibodies targeting different immunogens could further shed light on the attributes of TMEM-ir material and the biological relevance of TMEM106B fibril accumulation *in vivo*. Therefore, we generated and validated five polyclonal antibodies against distinct CTF immunogens, namely the residues 140-163, 164-187, 188-211, 239-250, and 253-274. The antibody recognizing the residues 239-250 (antibody no. 5: 239-250) was employed to identify cases positive for TMEM-ir material. Among the remaining four antibodies, antibody no. 3: 188-211 exhibited significant immunoreactivity in TMEM-ir material-positive cases. Comparative analyzes indicated that antibody no. 3: 188-211 and antibody no. 5: 239-250 likely recognized the same TMEM-ir material. The TMEM-ir material detected by antibody no. 3: 188-211 was observed across multiple brain cell types without co-localization with other pathogenic proteins. In conclusion, our findings suggest that the antibody recognizing the residues 188-211 displays immunohistochemical reactivity to TMEM-ir material. Therefore, in addition to the established antibody recognizing the residues 239-250, the antibody recognizing the residues 188-211 can potentially be used in immunohistochemical studies to further elucidate the significance of CTF accumulation in the brain.

## Introduction

Recent cryo-electron microscopy (cryo-EM) studies have revealed the accumulation of C-terminal fragments (CTF) (residues 120-254/274) of transmembrane protein 106B (TMEM106B) as amyloid fibrils in the sarkosyl-insoluble fractions of postmortem brain tissue from elderly individuals and patients with neurodegenerative diseases (Chang et al., 2022; Jiang et al., 2022; Schweighauser et al., 2022). Initially, no antibodies were available to detect CTF using immunohistochemistry (IHC). However, subsequent investigations demonstrated that a rabbit polyclonal antibody raised against residues 239-250 of TMEM106B could detect CTF in various states of aggregation, assembly, and solubility by IHC (Perneel et al., 2023; Vicente et al., 2023). In one of these studies, the regions positively stained with antibodies against residues 239-250 were referred to as TMEM106B C-terminal immunoreactive (TMEM-ir) material (Perneel et al., 2023).

The accumulation of TMEM-ir material in IHC has been consistently observed in elderly individuals and patients with neurodegenerative diseases, particularly those with frontotemporal dementia (FTD) caused by progranulin mutations, which is a risk factor for FTD (Finch et al., 2011). Furthermore, individuals with the two protective TMEM106B haplotypes of a coding variant of TMEM106B (Thr185Ser, encoded by single-nucleotide polymorphism (SNP) rs3173615) exhibit low levels of TMEM-ir material compared with individuals with the two risk TMEM106B haplotypes (Nicholson et al., 2013; Vicente et al., 2023). Additionally, limbic-predominant age-related TAR DNA-binding protein 43 (TDP-43) proteinopathy is associated with abundant TMEM-ir material (Neumann et al., 2023). In contrast, TMEM-ir material is absent in the brains of young subjects (Chang et al., 2022; Jiang et al., 2022; Schweighauser et al., 2022; Perneel et al., 2023; Vicente et al., 2023). As such, the antibody raised against residues 239-250 has significantly contributed to clarifying the characteristics of CTF in IHC. Interestingly, residues 239-250 are positioned at the center of the fibril core of the CTF. Thus, it is possible that residues 140-211, located on the periphery of the fibril core, are more easily recognizable by antibodies. In addition, residues 253-274, which are not part of the fibril core but may form an accessible fuzzy coat, may also be more easily recognized by antibodies (Jiang et al., 2022). Most importantly, immunohistochemical characterization of CTF using antibodies targeting different immunogens could help further characterize TMEM-ir material, thereby advancing our understanding of the biological relevance of TMEM106B fibril accumulation *in vivo*.

To this end, we explored the histological landscape of TMEM106B-immunoreactivities using multiple antibodies targeting diverse CTF immunogens. We found that, in addition to the previously reported TMEM106B antibody, which recognizes residues 239-250 of TMEM106B, a polyclonal antibody recognizing residues 188-211 of TMEM106B (antibody no. 3: 188-211) exhibits immunohistochemical reactivity to the TMEM106B CTF in the aging and disease-associated brain. Thus, antibody recognizing residues 188-211 can be considered for immunohistochemical investigations aiming at further elucidating the significance of CTF accumulation in the brain.

## Materials and methods

### Ethical considerations and subjects studied

This study was approved by the Institutional Review Board of Hiroshima University. The human samples were obtained from pathological autopsies conducted at Hiroshima University. Written informed consent was obtained from the subjects’ family members. The samples were anonymized, and personal information was dissociated from the test results. Data were analyzed anonymously, and all neuropathological procedures and analyzes were conducted in adherence to the ethical principles outlined in the Declaration of Helsinki. Immediately after autopsy, the specimens were fixed in 10% formalin for 3 weeks, and 7-μm-thick paraffin-embedded sections of frontal lobe were used for immunohistochemical staining. Table 1 summarizes the clinical features of the 11 subjects. Accumulation of TMEM-ir material has been consistently observed in elderly subjects and subjects with neurodegenerative diseases, particularly those with FTD caused by progranulin mutations. Thus, we recruited subjects with neurodegenerative diseases as follows: Cases 1–4, frontotemporal lobar degeneration with motor neuron disease; Case 6, dementia with Lewy bodies; and Case 7, multiple system atrophy. In addition, elderly subjects without neurodegenerative diseases were recruited as follows: Case 5, HTLV-1 associated myelopathy; and Case 8, intracranial hemorrhage. Furthermore, previous studies have demonstrated the absence of TMEM-ir material in young cases with or without degenerative disease (Chang et al., 2022; Jiang et al., 2022; Schweighauser et al., 2022; Perneel et al., 2023; Vicente et al., 2023). Therefore, we also recruited three subjects in their 20s, referred to as Cases 9–11.

**Table 1:** Cases included in this study.

Case number	Age (years)	Sex	Diagnose
Case 1	78	M	FTLD-MND
Case 2	72	F	FTLD-MND
Case 3	74	F	FTLD-MND
Case 4	74	F	FTLD-MND
Case 5	66	M	HAM
Case 6	86	M	DLB
Case 7	72	F	MSA
Case 8	80	M	ICH
Case 9	29	F	NMO
Case 10	26	F	HSAN type IV
Case 11	25	M	AIDP

FTLD-MND, frontotemporal lobar degeneration with motor neuron disease; HAM, HTLV-1 associated myelopathy; DLB, dementia with Lewy bodies; MSA, multiple system atrophy; ICH, intracranial hemorrhage; NMO, neuromyelitis optica; HSAN type IV, hereditary sensory and autonomic neuropathy type IV; AIDP, acute inflammatory demyelinating polyradiculoneuropathy.

### Antibodies

We obtained five purified antibodies (no. 1: 140-163, no. 2: 164-187, no. 3: 188-211, no. 4: 253-274, no. 5: 239-250) from rabbits injected with synthetic peptides corresponding to the C-terminal residues 140-163, 164-187, 188-211, 253-274, and 239-250 of TMEM106B, respectively. Cosmo Bio (Sapporo, Japan) was responsible for the antibody generation process. Briefly, the peptides were synthesized utilizing Fmoc solid-phase synthesis. The N-terminal of each peptide contained cysteine, and the C-terminal of each peptide was amidated. A comprehensive characterization of the synthetic peptides was performed through high-performance liquid chromatography (HPLC) and mass spectrometry (MS). The synthetic peptides were conjugated to keyhole limpet hemocyanin (KLH) through m-maleimidobenzoyl-N-hydroxysuccinimide ester (MBS) cross-linking, strategically targeting free sulfhydryl groups. This KLH-conjugated peptide complex served as the immunogen for rabbit immunization, a process that involved four successive immunizations. The initial immunization employed Complete Freund’s adjuvant, with the three subsequent immunizations utilizing incomplete Freund’s adjuvant. Following a 49-day incubation period after the first immunization, rabbit serum was collected and subjected to antibody purification utilizing affinity chromatography with peptide antigens. Each batch of rabbit serum was introduced to a peptide column and rotated for 30 min at room temperature (RT). After thorough washing with phosphate-buffered saline (PBS), antibody elution was achieved through the introduction of 3 M MgCl_2_. The elution process continued until the optical density at 280 nm dropped below 0.05. For final purification, the eluted antibodies underwent further processing using 14-kDa molecular weight cut-off (MWCO) dialysis membranes. Antibody concentrations were quantified through absorbance measurements at 280 nm.

### Enzyme-linked immunosorbent assay (ELISA)

The peptide antigens were diluted to a concentration of 10 μM in a binding buffer and dispensed into a 96-well plate before overnight incubation at 37°C. Subsequently, after washing three times with PBS, each well was treated with PBS containing 1% gelatin and incubated for 2 h at 37°C, followed by three additional washes with PBS containing 0.02% Tween. The purified antibodies were diluted in PBS supplemented with 0.1% Tween and 1% Bovine serum albumin (BSA), spanning dilution factors from 250-fold to 256,000-fold, and subsequently added to each well. After an incubation period of 1 h at RT, the wells were washed three times with PBS. Subsequently, a 1:15,000 dilution of an HRP-conjugated anti-IgG detection antibody (A120-111AP; Bethyl Laboratories) in PBS was added to each well, followed by a 30-min incubation at RT. Three final washes were performed using PBS containing 0.02% Tween and a reaction was initiated by adding 100 μL of p-Nitrophenylphosphate (PNPP) substrates (0201-01; Southern) to each well. The reaction mixture was then incubated for 30 min at RT. The reaction was halted by the addition of 50 μL of 3.5 N NaOH, and absorbance measurements were performed at 405 nm.

### Immunohistochemistry

Except for antibody no. 1: 140-163, each antibody was tested using IHC with various antigen retrieval methods, antibody concentrations, and amplification steps. Ultimately, formic acid (FA) was the most effective antigen retrieval agent. The following primary antibodies were used for IHC: no. 2: 164-187 (1:50), no. 3: 188-211 (1:500), no. 4: 253-274 (1:500), and no. 5: 239-250 (1:500). An antibody raised against the N-terminal domain of TMEM106B (N-terminal antibody) (Rabbit anti-TMEM106B Antibody, 1:1000; Bethyl Laboratories, Catalog # A303-439A), which recognizes the physiological form of TMEM106B (Schweighauser et al., 2022), was also used. The 7-μm-thick paraffin sections were deparaffinized and rehydrated. For antigen retrieval, FA treatment was performed for 1 min, followed by washing in distilled water for 3 min. The deparaffinized sections were subsequently incubated with 3% hydrogen peroxide in phosphate-buffered saline (PBS) for 30 min to eliminate endogenous peroxidase activity. After washing with PBS, each section was incubated with a blocking solution (Protein Block Serum-Free Ready-to-use Code X0909; Dako) for 20 min. Subsequently, each section was incubated with primary antibodies overnight at 4°C. After washing in PBS, antibody detection was performed using an EnVision system (EnVision+ System-HRP-labeled Polymer Anti-Rabbit, #K4003, Dako). Peroxidase labeling was visualized using diaminobenzidine (Dako). All sections were counterstained with hematoxylin.

### Double-label immunofluorescence

To determine whether TMEM-ir material co-localized with disease-specific pathological inclusions, we conducted double-label immunofluorescence (IF) using antibody no. 3: 188-211 and no. 5: 239-250 in combination with antibodies that recognize phospho-TDP-43 (mouse anti-phospho-TDP-43 Antibody, 1:1000; Cosmo bio, catalog # TIP-PTD-M01) or phospho-α-synuclein (mouse anti-phospho-α-Synuclein Antibody, 1:2000; Fujifilm, clone No. 64, catalog # 015-25,191). The secondary antibodies used were Alexa Fluor 568-or Alexa Fluor 488-conjugated anti-mouse IgG (H + L) or anti-rabbit IgG (H + L) (Invitrogen, 1:500), depending on the primary antibodies. Hoechst 33342 (Cellstain Hoechst 33342 solution; Dojindo) was used for nuclear counterstaining. Sections were treated with Sudan black to reduce autofluorescence. The immunostained preparations were examined under a fluorescence microscope (BZ-X710; Keyence).

To confirm the cell types in which TMEM-ir material accumulated, we conducted double-label IF using antibodies no. 3: 188-211 and no. 5: 239-250 in combination with markers for neurons (mouse anti-NeuN antibody, 1:100, Millipore, #MAB377), astrocytes (mouse anti-GFAP (GA5) antibody, 1:100, Cell Signaling, #3670), oligodendrocytes (mouse anti-Sox10 antibody, 1:100, abcam, catalog #ab216020), microglia (mouse anti-CD68 antibody, 1:100, DAKO, #M0814), and vascular endothelial cells (mouse anti-CD31 antibody, 1:20, abcam, # ab9498). Tyramide Signal Amplification (goat anti-mouse IgG and Alexa Fluor™ 488 Tyramide, Invitrogen, catalog # T20912) was used for these cell markers.

### Mirror imaging experiments

To investigate whether antibodies no. 3: 188-211 and no. 5: 239-250 recognize the same region in IHC, we prepared consecutive sections of the frontal lobe using a mirror technique. Briefly, two consecutive 2.5-μm-thick paraffin sections were placed on glass slides with their surfaces facing upward, allowing the sectioned surfaces of the same region to be stained with two different antibodies.

### Adsorption test

Adsorption tests were performed by IHC to verify the specificity of the antibodies. Antibodies no. 3: 188-211 and no. 5: 239-250 were pre-adsorbed overnight with 0 or 30 μg of the peptide immunogens used for their generation (the synthetic peptides corresponding to residues 188-211 and 239-250, respectively). These pellets were separated by centrifugation at 30,000 × g for 30 min. The supernatant was used for IHC following the procedure described above.

### Evaluation of TMEM106B C-terminal immunoreactive material

Adjacent sections of each subject were screened for the presence of TMEM-ir material using the antibodies no. 2: 164-187, no. 3: 188-211, no. 4: 253-274, and no. 5: 239-250. An N-terminal antibody was also used to recognize the physiological form of TMEM106B. Ten different standard 40 × microscopic fields (103,823 μm^2^) were randomly selected from each section using an Olympus DP74 microscope (Olympus Corporation). The overall amount of staining was graded quantitatively using the cellSens Dimension Desktop software (Olympus Corporation), based on the positive areas in standard 40 × microscopic fields. In addition, to evaluate the degree of concordance between the areas positively stained by antibody no. 3: 188-211 and those stained by antibody no. 5: 239-250, 15 corresponding standard 10 × microscopic fields (1,661,174 μm^2^) were selected from two consecutive 2.5-μm-thick paraffin sections with their surfaces facing upward stained with the antibodies no. 3: 188-211 and no. 5: 239-250. Pearson correlation coefficient was used to evaluate the correlation between the immunoreactivity of these two antibodies.

## Results

### Generation of antibodies targeting diverse C-terminal fragment (CTF) immunogens

**Figure 1:**
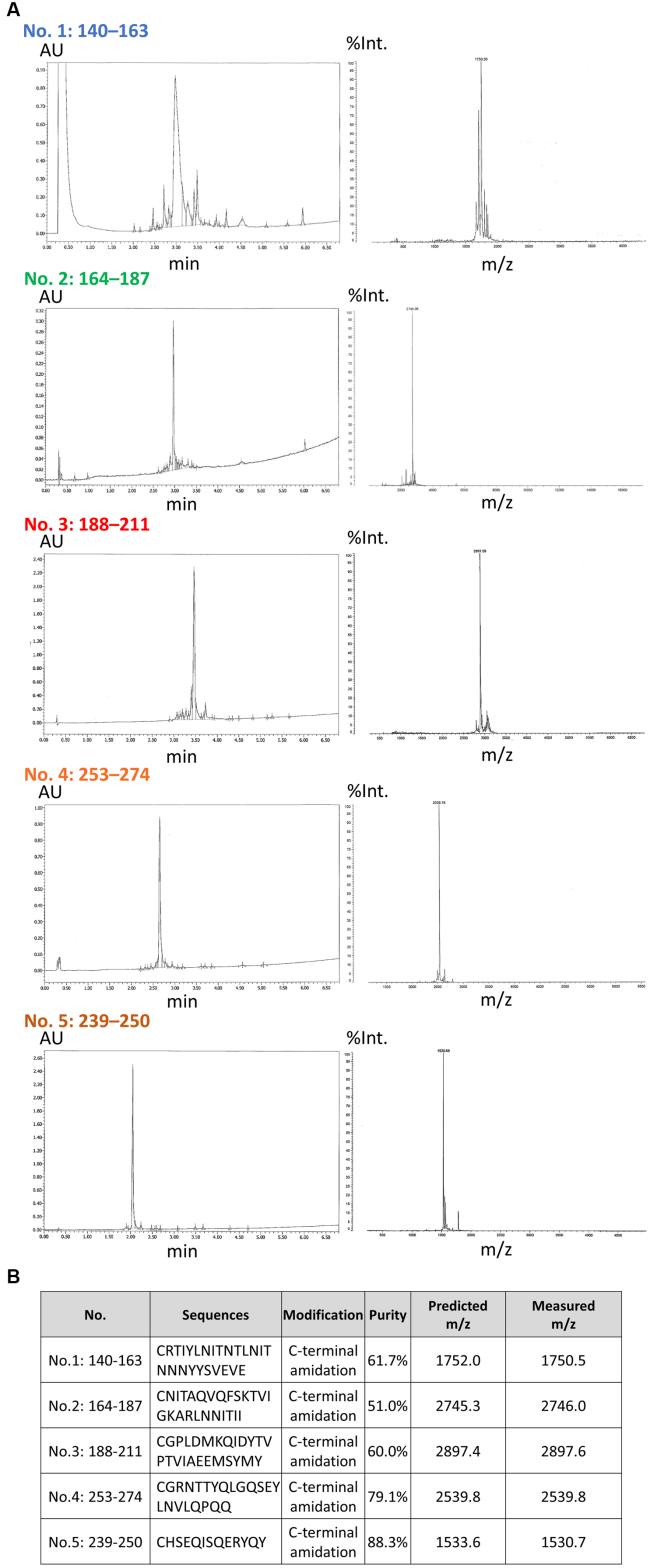
High-performance liquid chromatography and mass spectrometry analysis of synthetic peptides and properties of each antibody. **(A)** The results of a comprehensive analysis of the synthetic peptides with high-performance liquid chromatography (HPLC) and mass spectrometry (MS) are shown. The HPLC analysis was conducted to assess the purity of each synthesized peptide. Subsequently, the MS analysis was used to evaluate the quality attributes of the individual peptides. In the HPLC results (left), the horizontal axis is indicative of the elution time (min), while the vertical axis corresponds to the absorbance units (AU). In the MS results (right), the horizontal axis represents the mass-to-charge ratio (m/z) and the vertical axis the relative intensity (%Int.). **(B)** The properties of each peptide are shown. The N-terminal of each peptide contained cysteine, and the C-terminal of each peptide was amidated. The measured purities and m/z are shown.

**Figure 2:**
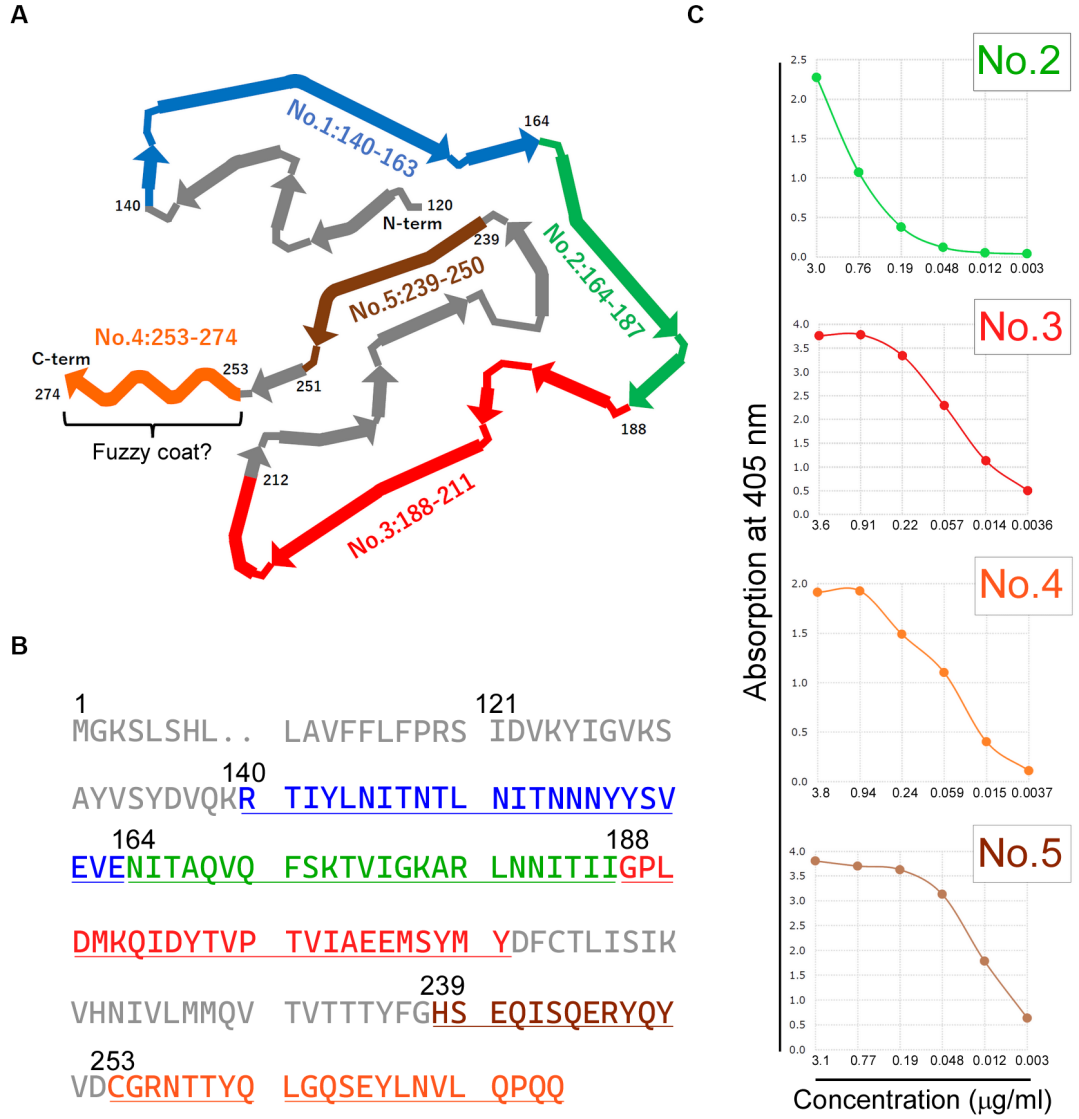
Generation and enzyme-linked immunosorbent assay (ELISA) Validation of antibodies targeting diverse C-terminal Fragment (CTF) Immunogens. **(A)** The schematic diagram illustrating the CTF of transmembrane protein 106B (TMEM106B), with each antibody’s specific recognition region indicated by colored regions. The diagram has been adapted and reformatted from Jiang et al. (2022). Residues 253–274 correspond to the potential existence of a fuzzy coat. **(B)** The residues of the peptide immunogens corresponding to each antibody are displayed in colors on the amino acid sequence of human TMEM106B (RefSeq Protein ID: NP_001127704.1). **(C)** The ELISA titer of each antibody is shown, excluding antibody no. 1: 140–163. ELISA validation revealed sufficient antibody titers for antibodies no. 2: 164–187, no. 3: 188–211, no. 4: 253–274, and no. 5: 239–250. The horizontal axis represents antibody concentration (μg/ml), whereas the vertical axis represents absorption at 405 nm. The colors used in each panel **(A–C)** represent the respective antibodies: *blue* for antibody no. 1: 140-163, *green* for antibody no. 2: 164-187, *red* for antibody no. 3: 188-211, *orange* for antibody no. 4: 253-274, and *brown* for antibody no. 5: 239-250.

To generate a panel of polyclonal antibodies that specifically targeted various CTFs of TMEM106B, we utilized purified antibodies from rabbits injected with synthetic peptides in this study. We first conducted a comprehensive characterization of the synthetic peptides through HPLC and MS analysis. The HPLC analysis aimed to assess the purity of each synthesized peptide, and it demonstrated that the purity of each synthesized peptide met the necessary criteria. Subsequently, MS analysis was employed to evaluate the quality attributes of the individual peptides. It revealed that the measured mass-to-charge ratio (m/z) was in close proximity to the predicted m/z (Figure 1). As a result, we concluded that the synthetic peptides were suitable for generating antibodies. Following the characterization of the synthetic peptides, we proceeded to generate a panel of polyclonal antibodies that specifically targeted various CTFs of TMEM106B. Five antibodies, referred to as antibodies no. 1: 140-163, no. 2: 164-187, no. 3: 188-211, no. 4: 253-274, and no. 5: 239-250, were isolated (Figures 2A,B). Subsequent ELISA validation revealed that these antibodies (no. 2: 164-187, no. 3: 188-211, no. 4: 253-274, and no. 5: 239-250) displayed sufficient antibody titers, indicating their ability to recognize the peptide immunogens used for their generation (Figure 2C). Conversely, antibody no. 1: 140-163 consistently exhibited low titers, despite repeated attempts using various immunized rabbits (data not shown). Consequently, the four antibodies (no. 2: 164-187, no. 3: 188-211, no. 4: 253-274, and no. 5: 239-250) with adequate titers were selected for downstream analyzes. The concentrations of antibodies no. 2: 164-187, no. 3: 188-211, no. 4: 253-274, and no. 5: 239-250 were 0.76 mg/mL, 0.91 mg/mL, 0.94 mg/mL, and at 0.77 mg/mL, respectively.

### Identification of subjects with abundant TMEM106B C-terminal immunoreactive (TMEM-ir) material in the brain

**Figure 3:**
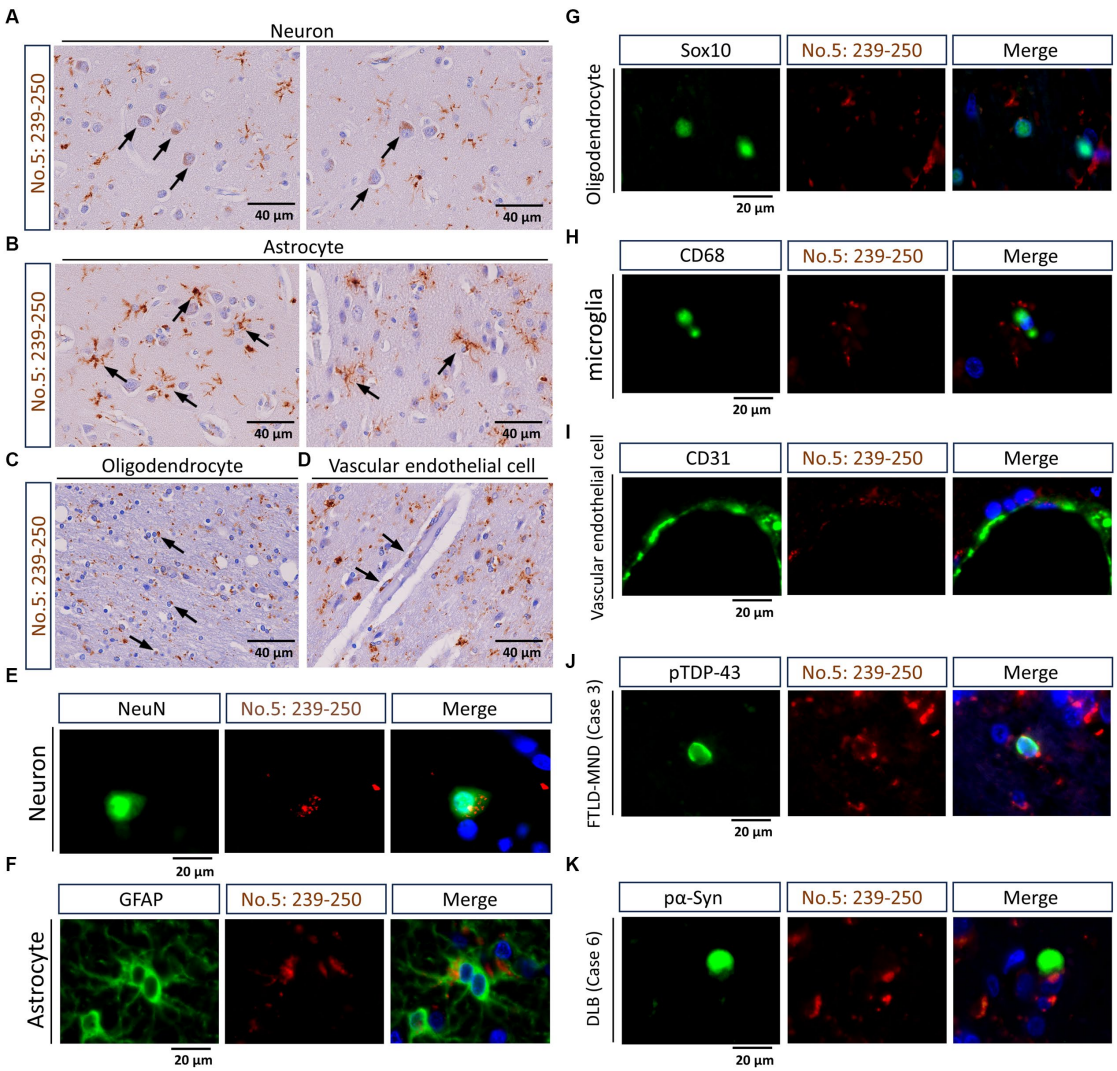
Staining characteristics of transmembrane protein 106B (TMEM106B) C-terminal immunoreactive (TMEM-ir) material by antibody no. 5: 239-250. **(A–D)** Representative cytoplasmic staining patterns of antibody no. 5: 239-250 in frontal lobe sections from TMEM-ir material-positive cases are shown. The image was obtained from Case 3 with frontotemporal lobar degeneration with motor neuron disease (FTLD-MND). Cells positive for cytoplasmic TMEM-ir material as stained by antibody no. 5: 239-250 exhibited morphological features of neurons **(A)**, astrocytes **(B)**, oligodendrocytes **(C)**, and vascular endothelial cells **(D)**. Scale bar: 40 μm. **(E–I)** Representative double-label immunofluorescence (IF) staining images performed using antibody no. 5: 239-250 [*red*, **(E–I)**] in combination with markers for neurons (NeuN) [*green*, **(E–I)**] astrocytes (GFAP) [*green*, **(F)**], oligodendrocytes (Sox10) [*green*, **(G)**], microglia (CD68) [*green*, **(H)**], and vascular endothelial cells (CD31) [*green*, **(I)**]. Images were obtained from Case 3 [FTLD-MND, **(E–I)**]. Nuclear staining was performed with Hoechst [*blue*, **(E–I)**]. Scale bar: 20 μm. **(J,K)** Representative double-label IF staining images performed using antibody no. 5: 239-250 [*red*, **(J,K)**] in combination with antibodies against phospho-TDP-43 (pTDP-43) [*green*, **(J)**] or phospho-α-synuclein (pα-Syn) [*green*, **(K)**]. Images were obtained from Case 3 [FTLD-MND, **(J)**] and Case 6 [dementia with Lewy bodies, **(K)**]. The TMEM-ir material stained by antibody no. 5: 239-250 did not co-localize with pTDP-43 or pα-Syn staining. Nuclear staining was performed with Hoechst [*blue*, **(J,K)**]. Scale bar: 20 μm.

Using IHC and double-label IF, we confirmed that the antibody targeting the residues 239-250 of TMEM106B has extensive detection capabilities for TMEM-ir material (Perneel et al., 2023; Vicente et al., 2023). Specifically, in IHC, TMEM-ir material stained by antibody no. 5: 239-250 was observed in the cytoplasm of various cell types in the frontal lobe. Moreover, in double-label IF, TMEM-ir material stained by antibody no. 5: 239-250 accumulated in various cell types without colocalization with other pathogenic proteins (Figure 3). Considering the extensive detection capabilities of the antibody targeting the residues 239-250 of TMEM106B for TMEM-ir material in individuals with TMEM106B fibril accumulation (Perneel et al., 2023; Vicente et al., 2023), we utilized antibody no. 5: 239-250 to assess the presence of TMEM-ir material in eight subjects aged >65 years (corresponding to Cases 1-8).

**Figure 4:**
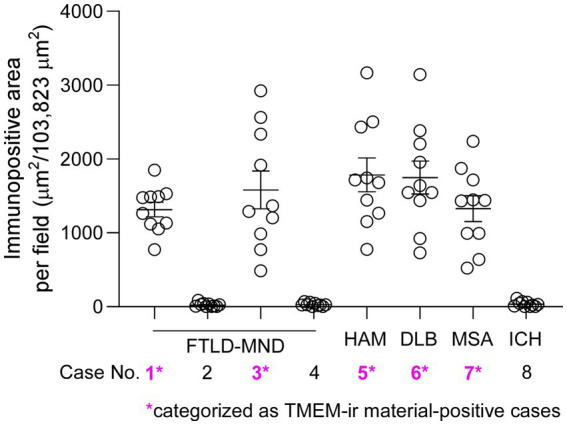
Recruitment of subjects with abundant TMEM106B C-terminal immunoreactive (TMEM-ir) material in the brain. Positive areas stained by antibody no. 5: 239-250 were quantified in frontal lobe sections from subjects aged >65 years (Cases 1–8). Ten different standard 40× microscopic fields were randomly selected and assessed within one section from each case. Cases 1, 3, 5, 6, and 7, which exhibited prominent and widespread positive areas, were classified as TMEM-ir material-positive (*pink*) and selected for further analyzes. Cases 2, 4, and 8, which displayed limited positive areas, were determined to be negative for TMEM-ir material. The vertical axis represents the immunopositive area per field (μm^2^/103,823 μm^2^). Each circle represents the result from one microscopic field. The error bars indicate the standard error of the mean.

The positive areas in ten different standard 40× microscopic fields for each case are depicted in Figure 4. Subjects in Cases 1, 3, 5, 6, and 7 exhibited conspicuous and widespread positive areas, whereas subjects in Cases 2, 4, and 8 displayed limited positive areas. Consequently, we concluded that TMEM-ir material was present in Cases 1, 3, 5, 6, and 7, which were categorized as TMEM-ir material-positive cases for further analyzes. In addition, we recruited three subjects in their 20s (Cases 9–11) as TMEM-ir material-negative cases, later confirming the absence of TMEM106B-ir material using antibody no. 5: 239-250 (data not shown).

### Validation of the immunoreactivities of antibodies targeting diverse CTF immunogens

**Figure 5:**
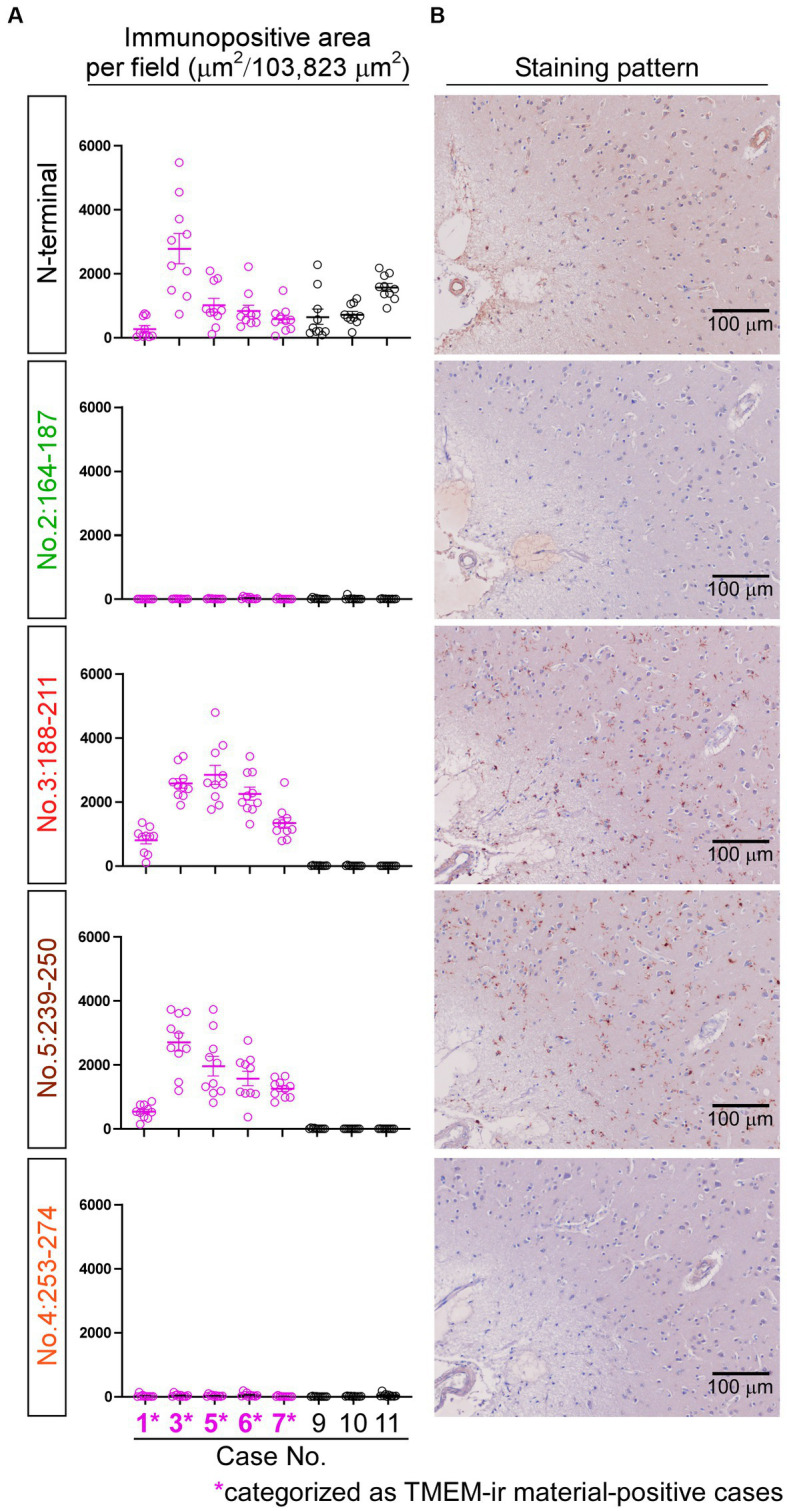
Immunoreactivities of antibodies targeting diverse C-terminal fragment immunogens. **(A)** The positive areas stained by each antibody were quantified in frontal lobe sections from TMEM-ir material-positive cases (*n* = 5, depicted in *pink*) and TMEM-ir material-negative cases (*n* = 3, depicted in *black*). Antibodies no. 3: 188-211 and no. 5: 239-250 exhibited larger positive areas in TMEM-ir material-positive cases compared with TMEM-ir material-negative cases. The N-terminal antibody showed similar positive staining areas in both TMEM-ir material-positive and TMEM-ir material-negative cases. The vertical axis represents the immunopositive area per field (μm^2^/103,823 μm^2^). Each circle represents the result from one microscopic field. The error bars indicate the standard error of the mean. **(B)** Representative staining patterns of each antibody in frontal lobe sections from TMEM-ir material-positive cases are shown (image obtained from Case 3 [frontotemporal lobar degeneration with motor neuron disease]). Antibodies no. 3: 188-211 and no. 5: 239-250 stained abundant TMEM-ir material, whereas the N-terminal antibody exhibited only diffuse cytoplasmic staining. Antibodies no. 2: 164-187 and no. 4: 253-274 displayed nonspecific staining patterns. Scale bar: 100 μm.

We utilized TMEM-ir material-positive (*n* = 5; Cases 1, 3, 5, 6, and 7) and TMEM-ir material-negative (*n* = 3; Cases 9–11) cases to validate the immunoreactivities of the three antibodies targeting diverse CTF immunogens (i.e., no. 2: 164-187, no. 3: 188-211, no. 4: 253-274). Among these, staining with antibodies no. 3: 188-211 exhibited larger positive areas in TMEM-ir material-positive cases than in TMEM-ir material-negative cases, suggesting a significant affinity of this antibody to TMEM-ir material. Both TMEM-ir material-positive and TMEM-ir material-negative cases showed similar positive staining areas with the N-terminal antibody, suggesting no significant difference in the expression of the physiological TMEM106B protein (Figure 5A). The staining patterns of each antibody are shown in Figure 5B. Antibodies no. 3: 188-211 and no. 5: 239-250 stained abundant TMEM-ir material, whereas the N-terminal antibody exhibited only diffuse cytoplasmic staining. The staining patterns of antibodies no. 2: 164-187 and no. 4: 253-274 were likely nonspecific. Given its significant affinity for TMEM-ir material, antibody no 3: 188-211 was chosen for downstream analyzes.

### Mirror imaging experiments and adsorption test for antibodies no. 3: 188-211 and no. 5: 239-250

**Figure 6:**
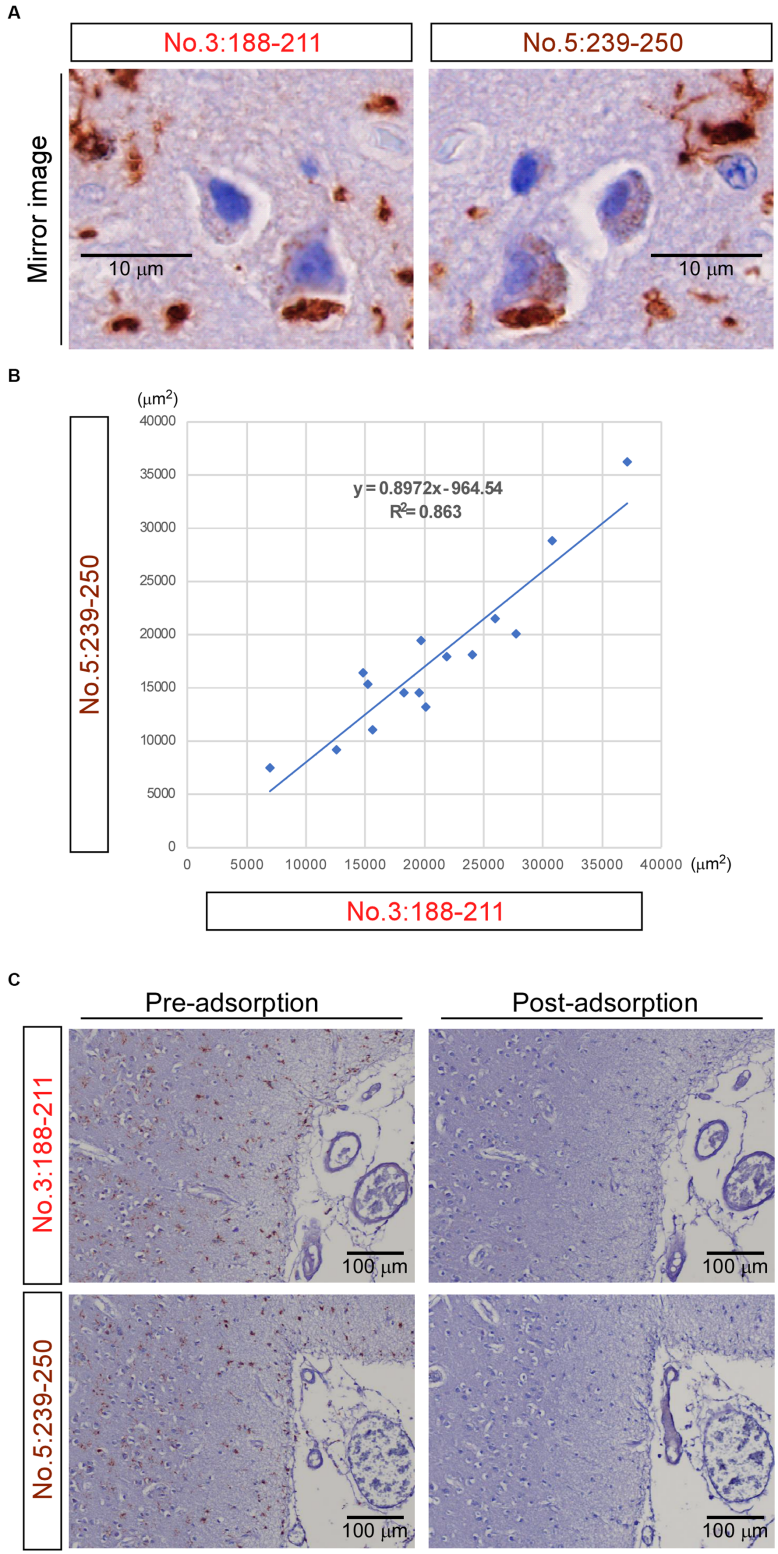
Mirror imaging experiments and adsorption test. **(A)** Mirror imaging experiments: adjacent frontal lobe sections from Case 3 (frontotemporal lobar degeneration with motor neuron disease) were symmetrically mounted on glass slides and stained with antibodies no. 3: 188-211 and no. 5: 239-250, respectively. The images indicated that antibodies no. 3: 188-211 and no. 5: 239-250 likely recognize the same transmembrane protein 106B (TMEM106B) C-terminal immunoreactive material. Scale bar: 10 μm. **(B)** The degree of concordance between the areas positively stained by antibody no. 3: 188–211 and those stained by antibody no. 5: 239-250. The Pearson correlation coefficient (r) was 0.929. Each dot represents the positive areas (μm^2^) of the corresponding same standard 10 × microscopic fields of two consecutive 2.5-μm-thick paraffin sections with their surfaces facing upward stained by these two antibodies. The solid line in the graph represents the regression line. **(C)** In the adsorption test, adjacent frontal lobe sections from Case 3 were mounted on glass slides in the same direction and stained with antibody no. 3: 188-211 and antibody no. 5: 239-250, respectively. When these two antibodies were pre-adsorbed overnight with 0 μg of the peptide immunogens, they stained abundant TMEM-ir material (pre-adsorption). When these two antibodies were pre-adsorbed overnight with 30 μg of the peptide immunogens, no staining was observed (post-adsorption), indicating their specific immunohistochemical reactivity to the C-terminal fragments. Scale bar: 100 μm.

Mirror imaging experiments were conducted to investigate whether antibodies no. 3: 188-211 and no. 5: 239-250 recognize the same material in IHC. The images obtained from the experiments indicated that antibodies no. 3: 188-211 and no. 5: 239-250 likely recognize the same TMEM-ir material (Figure 6A). The corresponding standard 10 × microscopic fields of two consecutive 2.5-μm-thick paraffin sections with their surfaces facing upward stained by these two antibodies were evaluated. Strong positive correlations were observed from the areas positively stained by antibody no. 3: 188-211 and those stained by antibody no. 5: 239-250 (*r* = 0.929) (Figure 6B).

In the adsorption test, antibodies no. 3: 188-211 and no. 5: 239-250 were pre-adsorbed overnight with 0 μg of the peptide immunogens. Consequently, they stained for abundant TMEM-ir material. Conversely, when the two antibodies were pre-adsorbed overnight with 30 μg of the peptide immunogens, no staining was observed (Figure 6C). Taken together, these findings suggest that the polyclonal antibody recognizing residues 188-211 and 239-250 of TMEM106B demonstrates immunohistochemical reactivity to the TMEM106B CTF with comparable sensitivity and specificity.

### Staining characteristics of TMEM106B C-terminal Immunoreactive material by antibody no. 3: 188-211

**Figure 7:**
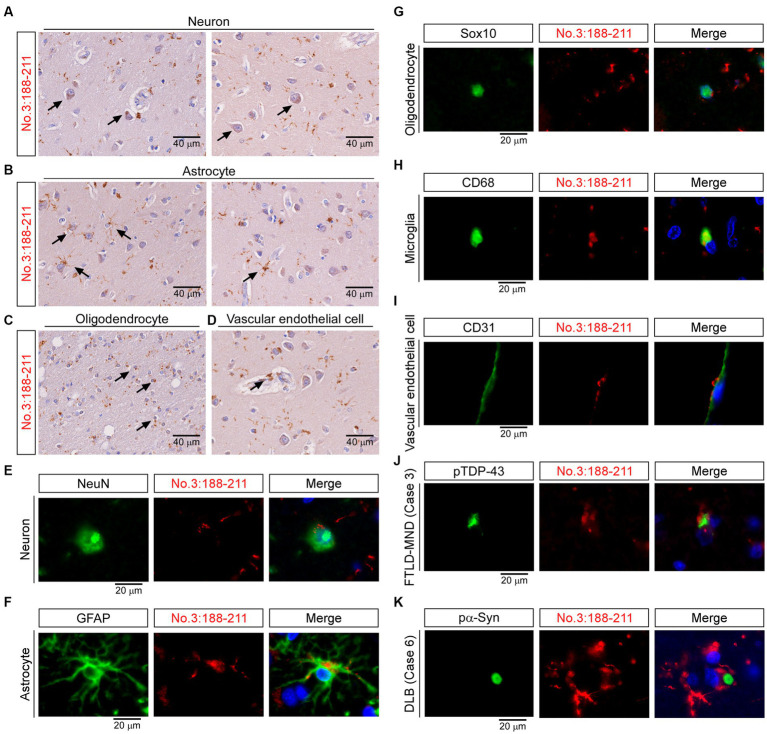
Staining characteristics of transmembrane protein 106B (TMEM106B) C-terminal immunoreactive (TMEM-ir) material by antibody no. 3: 188-211. **(A–D)** Representative cytoplasmic staining patterns of antibody no. 3: 188-211 in frontal lobe sections from TMEM-ir material-positive cases are shown (image obtained from Case 3 [frontotemporal lobar degeneration with motor neuron disease {FTLD-MND}]). Cells positive for cytoplasmic TMEM-ir material as stained by antibody no. 3: 188-211 exhibited morphological features of neurons **(A)**, astrocytes **(B)**, oligodendrocytes **(C)**, and vascular endothelial cells **(D)**. Scale bar: 40 μm. **(E–I)** Representative double-label immunofluorescence (IF) staining images performed using antibody no. 3: 188–211 [*red*, **(E–I)**] in combination with markers for neurons (NeuN) [*green*, **(E)**], astrocytes (GFAP) [*green*, **(F)**], oligodendrocytes (Sox10) [*green*, **(G)**], microglia (CD68) [*green*, **(H)**] and vascular endothelial cells (CD31) [*green*, **(I)**]. Images were obtained from Case 3 [FTLD-MND, **(E–I)**]. Nuclear staining was performed with Hoechst [*blue*, **(E–I)**]. Scale bar: 20 μm. **(J,K)** Representative double-label IF staining images performed using antibody no. 3: 188–211 [*red*, **(J,K)**] in combination with antibodies against phospho-TDP-43 (pTDP-43) [*green*, **(J)**] or phospho-α-synuclein (pα-Syn) [*green*, **(K)**]. Images were obtained from Case 3 [FTLD-MND, **(J)**] and Case 6 [dementia with Lewy bodies, **(K)**]. The TMEM-ir material stained by antibody no. 3: 188–211 did not co-localize with pTDP-43 or pα-Syn. Nuclear staining was performed with Hoechst [*blue*, **(J,K)**]. Scale bar: 20 μm.

Finally, we studied the staining characteristics of the TMEM-ir material as detected by antibody no. 3: 188-211. In IHC, TMEM-ir material stained by antibody no. 3: 188-211 was observed in the cytoplasm of various cell types, including neurons, astrocytes, oligodendrocytes, and vascular endothelial cells in the frontal lobe (Figures 7A–D). Double-label IF analyzes also showed that TMEM-ir material stained by antibody no. 3: 188-211 accumulated in neurons, astrocytes, oligodendrocytes, microglia, and vascular endothelial cells (Case 3) (Figures 7E–I). In addition, TMEM-ir material stained by antibody no. 3: 188-211 did not co-localize with phospho-TDP-43 staining in a subject with frontotemporal lobar degeneration with motor neuron disease (Case 3) or phospho-α-synuclein staining in a subject with dementia with Lewy bodies (Case 6) (Figures 7J,K). Thus, we confirmed that the antibody targeting the residues 188-211 of TMEM106B has extensive detection capabilities for TMEM-ir material.

## Discussion

In this study, by using an antibody targeting the residues 239-250 of the TMEM106B protein as a reference antibody to identify TMEM-ir material, we demonstrated that polyclonal antibody no. 3: 188-211 exhibited notable immunohistochemical reactivity to the CTF of TMEM106B within aging and disease-associated brain tissues. In particular, antibody no. 3: 188-211 demonstrated a remarkable capacity to discriminate between cases displaying positive TMEM-ir material and those lacking such material. The TMEM-ir material detected by antibody no. 3: 188-211 accumulated across diverse cell types and exhibited no co-localization with the tested pathogenic proteins inclusions. Notably, the staining pattern of the TMEM-ir material recognized by antibody no. 3: 188-211 closely resembled that recognized by antibody no. 5: 239-250, and a high degree of concordance was observed between the areas positively stained by antibody no. 3: 188-211 and those stained by antibody no. 5: 239-250 in TMEM-ir material-positive cases. These observations reinforce the findings of previous studies regarding the characteristics of TMEM-ir material (Perneel et al., 2023; Vicente et al., 2023). Furthermore, our results suggest that antibody no. 3: 188-211 has a potential comparable to that of the reference antibody in targeting the residues 239-250 for detecting TMEM-ir material *in vivo* (Perneel et al., 2023; Vicente et al., 2023).

There are several limitations to this study. Firstly, the three-dimensional configuration of the antigen peptides used for rabbit immunization was not analyzed. Consequently, it is possible that the intended sequence may not functioned as expected due to antigen peptide aggregation *in vivo*. Therefore, the fact that the titers of antibodies no. 2: 164-187 and no. 4: 253-274 during the ELISA validation assays were found to be inferior to those of antibodies no. 3: 188-211 and no. 5: 239-250, and antibody no. 1: 140-163 consistently exhibited low titers does not necessarily imply that residues 140-163, 164-187, and 253-274 are inappropriate as potential antigenic sites in immunobiological assays. Even if it were possible to generate antibodies that bind to residues 140-163, 164-187, and 253-274 and if the TMEM-ir material contained the appropriate sequences, it is unclear whether these antibodies would be able to recognize the TMEM-ir material in paraffin-embedded sections by IHC. The TMEM-ir material was detected using antibodies no. 3: 188-211 and no. 5: 239-250, after antigen retrieval by FA, suggesting that at least some of the epitopes were exposed using this antigen retrieval method. However, it is unclear whether epitopes in the TMEM-ir material that recognize the residues 140-163, 164-187, and 253-274 are exposed by FA antigen retrieval; thus, other antigen retrieval methods may be necessary. Overall, our results suggest that it may be challenging to generate antibodies aimed at detecting epitopes in TMEM-ir material by IHC using standard peptide immunization methods with synthetic peptides corresponding to residues 140-163 and 164-187 situated at the fringes of the fibril core, or with synthetic peptides corresponding to residues 253-274 that may not be integral to the fibril core and could form an exposed fuzzy coat. Nevertheless, further efforts aimed at optimizing epitope presentation through immunization and using various antigen retrieval methods are crucial for evaluating the suitability of antibodies recognizing the residues 140-163, 164-187, 188-211, and 253-274 in detecting the TMEM-ir material.

Secondly, the distinction between TMEM-ir material-positive and-negative cases in this study was based on our immunohistochemical results using no. 5: 239-250. However, by definition, categorizing TMEM-ir material-positive cases necessitates cryo-EM elucidation (Chang et al., 2022; Jiang et al., 2022; Schweighauser et al., 2022). It is important to note that the antibody SB0051, used in a previous report (Perneel et al., 2023), differs from the no. 5: 239-250 used in the present study. Specifically, the two antibodies were generated from different rabbits, introducing a biological variable. Furthermore, owing to their differences and polyclonal nature, the antibodies may recognize different epitopes. A side-by-side comparison of antibody recognizing residues 188-211 and SB0051, performed using brain samples analyzed by cryo-EM electron microscopy, is required to fully clarify the performance of antibodies recognizing residues 188-211. Thirdly, there is a lack of clarity regarding the use of TMEM106B genetic polymorphism for designating FTLD-MND cases as TMEM-ir material-negative cases, and conversely, young, non-degenerative HAM cases as TMEM-ir material-positive cases (Jiao et al., 2023). Comprehensive studies involving cryo-EM observation, TMEM106B prototype evaluation, genetic polymorphism analysis, and the examination of a substantial number of cases are warranted for resolving these ambiguities.

## Conclusion

The antibody recognizing the residues 188-211 offers potential in immunohistochemical studies aiming to further elucidate the significance of CTF accumulation in the brain.

## Data availability statement

The raw data supporting the conclusions of this article will be made available by the authors, without undue reservation.

## Ethics statement

The studies involving humans were approved by Institutional Review Board of Hiroshima University. The studies were conducted in accordance with the local legislation and institutional requirements. The participants provided their written informed consent to participate in this study.

## Author contributions

RI: methodology, investigation, formal analysis, and writing – original draft preparation. YY: conceptualization, methodology, and writing – review and editing. MN and TT: methodology and writing – review and editing. HM: methodology, writing – review and editing, and supervision. All authors contributed to the article and approved the submitted version.
